# Effects of intramuscular alfaxalone and dexmedetomidine alone and combined on ocular, electroretinographic, and cardiorespiratory parameters in normal cats

**DOI:** 10.3389/fvets.2024.1407928

**Published:** 2024-07-03

**Authors:** Yizhe Guo, Sichao Mao, Zhenlei Zhou

**Affiliations:** College of Veterinary Medicine, Nanjing Agricultural University, Nanjing, China

**Keywords:** alfaxalone, dexmedetomidine, electroretinography, intraocular pressure, cats

## Abstract

**Background:**

This study aimed to determine the effects of intramuscular (IM) administration of alfaxalone with or without dexmedetomidine on short electroretinography (ERG), ocular parameters and cardiorespiratory in healthy cats.

**Methods:**

Eight healthy female spayed cats were treated with three sedation protocols: IM administration of 5 μg/kg dexmedetomidine (DEX), 5 mg/kg alfaxalone (ALF), and 5 μg/kg dexmedetomidine plus 5 mg/kg alfaxalone (DEX + ALF). The washout period after each treatment was 2 weeks. Physiological parameters, time metrics, intraocular pressure (IOP), Schirmer tear test 1 (STT-1) and a short ERG protocol were recorded. For age data, weight data, time metrics and ERG data, one-way ANOVA with Bonferroni posterior comparisons were performed. For physiological parameters, IOP and STT-1 data, two-way repeated measures ANOVA with Bonferroni posterior comparisons were performed. Statistical significance was set at a *p*-value <0.05.

**Results:**

IOPs were increased in all three groups compared to baseline and showed no significant differences among three groups at any time point. STT-1 values were decreased significantly during the process. Significant differences were noticed between a-wave amplitude in the dark-adapted response between DEX and ALF, and a-wave amplitude in light-adapted response between ALF and DEX + ALF.

**Conclusion:**

This study demonstrates the feasibility of three sedation protocols for short ERG recording in cats. All these treatments resulted in increased IOP values and reduced STT-1 values. But baseline data of ERG was not obtained as a blank control in cats.

## 1 Introduction

The electroretinogram (ERG) has been an important tool in clinical ophthalmology for evaluating retinal function since 1865 (1). It provides an invaluable mean of assessing retinal function with opaque media or during the early phases of diseases without significant funduscopic changes (2). Glaucoma is an ocular disorder characterized by increased intraocular pressure (IOP), and the rebound tonometry is a widely used method of IOP measurement (3). Aqueous tear deficiency (dry eye) can lead to serious ocular complications, and Schirmer tear test-1 (STT-1) examination is commonly used in clinical practice (4).

In addition to a quick retinal check performed in partially conscious animal, anesthesia is preferred during the ERG examination to prevent artifacts caused by involuntary eyelid twitch, body or globe movements as well as minimize stress induced by physical restraint (2, 5–7). The results of IOP and STT-1 will be affected by anesthesia, the majority of measurements are taken without anesthesia. Intraocular pressure (IOP) is primarily determined by aqueous humor dynamics and other factors including intraocular blood volume, central venous pressure, and extraocular muscle tone (8). Anesthetic drugs influence IOP mainly though extraocular muscles, affecting the success of ophthalmic procedures (9, 10). Previous studies reported an increase in IOP in cats administered with propofol (11). Moreover, reduced tear production is observed in cats with administration of medetomidine-ketamine (12). Corneal exposure and tear film evaporation during sedation may lead to ocular complications such as ulcerative keratitis (13). But sedation of less compliant animals provides ideal conditions for optimal ophthalmic examination and enhances safety for both the patient and examiner (14). Furthermore, general anesthesia carries inherent risks like cardiopulmonary depression (15). Thus, there is an urgent need for a simple, safe, and effective chemical restraint for short ERG recordings in cats.

Alfaxalone is a progesterone analog that have rapid onset and brief duration of action through γ-aminobutyric acid type A (GABAA) receptors (16–18). Compared to other anesthetic agents, alfaxalone offers advantages such as minimal cardiovascular depression in cats (19–21), dogs (22), and human (23). But alfaxalone is associated with certain side effects. Intravenous (IV) administration of alfaxalone at doses ranging from 0 to 50 mg/kg has been shown to induce dose-dependent suppressive effect on cardiorespiratory function and hyperkinesia during recovery in unpremedicated cats (21, 24). The most common adverse effects of alfaxalone administration in cats include apnea and hypoventilation (21). Dexmedetomidine is a sedative drug that commonly used in veterinary medicine. It is an alpha-2 adrenoceptor agonist witch exerts sedative effects through activation of central presynaptic and postsynaptic alpha-2 receptors in the locus coeruleus (25). Cats administered with alfaxalone (5 mg/kg) intramuscularly (IM) were deeply sedated, while cats administered with alfaxalone IM (5 mg/kg) combined with 20 and 40 μg/kg dexmedetomidine were anaesthetized (26). Induction with alfaxalone alone or in combination with dexmedetomidine intramuscularly (IM) is reported in cats, however, there is a lack of data specifically about their effects on the ERG, IOP, and STT-1 in cats (22).

We hypothesized that similar to anesthetic drugs such as propofol, IM administration of alfaxalone would increase IOP, decrease tear production, and have no effect on the ERG recordings in cats, while the use of dexmedetomidine could influence the ERG recordings and physiological variables. This study was designed to compare the effects of IM administration of alfaxalone alone or in combination with dexmedetomidine on the short ERG protocol, IOP, STT-1, sedative effect, reversal times, and physiological variables in cats.

## 2 Materials and methods

### 2.1 Animals

All procedures were performed following the guidelines of the Association for Research in Vision and Ophthalmology Statement for the Use of Animals in Ophthalmic and Vision Research. Eight spayed female cats were recruited for this study. They were housed individually in a room under controlled temperature (21°C) and light (7:00–19:00): dark (19:00–7:00 next day) cycle. Cats were socialized and adapted to human handling for 2 months prior to the study. Two weeks before study, the cats were specifically assimilated to tonometry. The animal study was approved by the Institutional Animal Care and Use Committee of Nanjing Agricultural University. The approval number was NJAU2022025.

The American Society of Anesthesiologists (ASA) classification was utilized in this study (27). Before the study, physical examination and ophthalmic examinations, including X-ray examination (InnoVet Versa DR Veterinary System; Radiology Imaging Solutions), echocardiography examination (W70 Vet; Esaote), complete blood count test (BC-5000 Vet; Mindray Animal Care), blood biochemistry test (Catalyst One Chemistry Analyzer; Idexx), blood gas analysis (i-STAT 1 analyzer; Abbott), electrocardiogram (ECG, BeneHeart R3A; Mindray), Schirmer tear test I (I-DEW Tear strips Schirmer Test; Ophtalmo), fluorescein staining (I-DEW FLO Fluorescein 1MG Strips; Ophtalmo), intraocular pressure with rebound tonometry setting as “do” (Tonovet; Icare Finland), slit lamp biomicroscopy (SL-17; Kowa), and fundus imaging system examination (Clear View 2; Menicon) were conducted on the cats. The inclusion criteria for cats included in this trial were that there are no abnormalities in the above examinations and the ASA classification was class I. All cats received an ASA class I assessment without ocular abnormalities.

### 2.2 Study design

**Figure 1:**
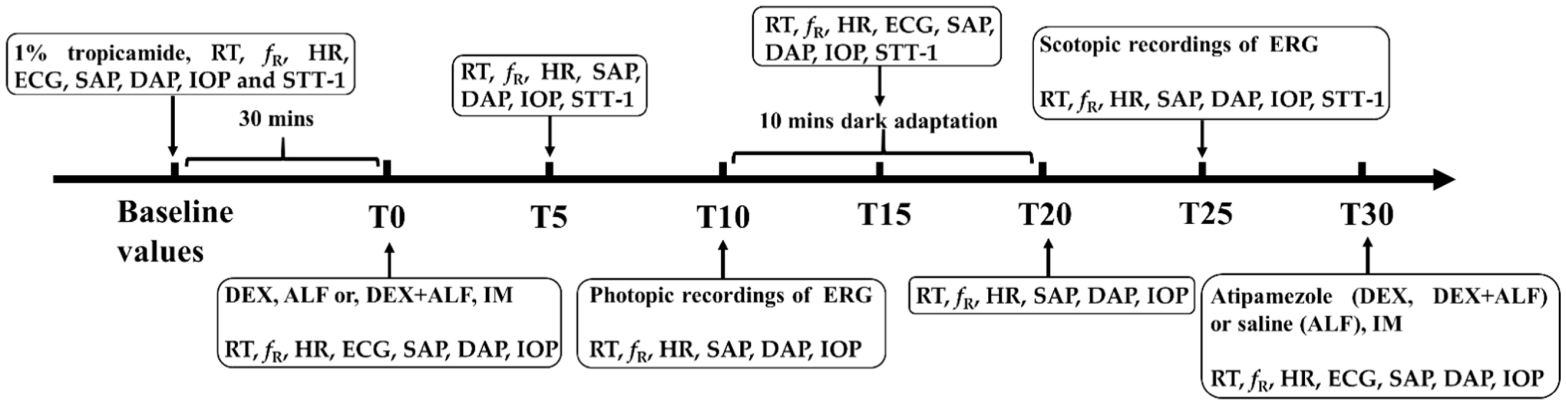
Flow chart of this study. Baseline values were measured 30 min before the administrated of drugs. T0 was the timepoint after administrated of drugs. DEX + ALF, the group received a combination of dexmedetomidine (5 μg/kg) and alfaxalone (5 mg/kg) intramuscularly (IM); DEX, the group received dexmedetomidine (5 μg/kg) intramuscularly; ALF, the group received alfaxalone (5 mg/kg) intramuscularly; IM, intramuscular injection, *n* = 8 cats; ERG, electroretinography; RT, rectal temperature; *f*_R_, respiratory rate; HR, heart rate; ECG, electrocardiogram; SAP, systolic arterial pressure; DAP, diastolic arterial pressure; IOP, the intraocular pressure; STT-1, Schirmer tear test 1.

The process of this study was shown in Figure 1. The right eye was treated with 5 mg/mL tropicamide (Tropicamide Eye Drops, Bausch + Lomb) to dilate the pupil 30 min before the injection of drugs. The cats were treated using three different protocols with a washout period of 2 weeks after each treatment. IM injection of 5 μg/kg dexmedetomidine (Dexdomitor, Orion Pharma) and 5 mg/kg alfaxalone (Alfaxan, Jurox Animal Health) was the DEX + ALF group; IM injection of 5 μg/kg dexmedetomidine was the DEX group; IM injection of 5 mg/kg alfaxalone was the ALF group. The drugs were mixed in one syringe. Sodium chloride 0.9% solution was administrated for DEX (0.5 mL/kg) and ALF (0.01 mL/kg) groups, respectively, to ensure a same amount of IM fluid. The time of drug administration was considered T0, with various interventions performed at 5 min intervals for 30 min (represented as T5, T10, T15, T20, T25, and T30) except ECG, STT-1 and ERG.

This study was performed between 15:00 and 17:00. The cats were fasted for 6 h before the experiment, but water was available until 1 h before the start of the experiment. A 22 G intravenous catheter was placed in the cephalic vein before sedation. The cats were positioned in sternal recumbency and received oxygen via a face mask (2 L/min). Body temperatures were preserved using an air blanket. The Lift-head time (LH), sternal recumbency time (SRr), and standing time (ST) were recorded to evaluate sedative effects of drugs during recovery from anesthesia (26). The LH was determined from drug injection to the first instance of the cat autonomously raising its head. The SRr was determined from drug injection to the first occurrence of the cat returning to the sternal recumbency. The ST was determined from drug injection to the first instance of the cat autonomously standing.

### 2.3 Physiological variables evaluations

Systolic arterial pressure (SAP), diastolic arterial pressure (DAP), rectal temperature (RT), heart rate (HR) and respiratory rate (*f*_R_) were measured at baseline and every 5 min from 0 to 30 min after treatment. The ECG was evaluated at baseline and every 15 min from 0 to 30 min after treatment. The SAP and the DAP was determined by a veterinary blood pressure monitor (SunTech Vet20; SunTech Medical) with a cuff width approximately 40% of the limb circumference positioned on the right forelimb (28). Three measurements were taken and the average was considered as the blood pressure for that time point. The RT was determined by a compact anesthesia monitor (S/5; GE Healthcare) using an intrarectal sensor. The HR and the ECG were determined by a lead II electrocardiogram (BeneHeart R3A; Mindray). The *f*_R_ was counted by observing chest movement during the respiratory cycle for a period of 30 s. All physiological variables evaluations, LH, SRr and ST were recorded by the same experienced veterinarian who was blinded to group allocation.

### 2.4 Ophthalmic examination

IOP and STT-1 were measured from the left eye of each cat in different groups. IOP and STT-1 examinations were performed by the same experienced veterinarian who was blinded to group allocation. IOP was registered at baseline and every 5 min from 0 to 30 min after treatment. STT-1 was registered at baseline and 5, 15, 25 min after treatment. IOP was recorded on the central cornea without topical anesthesia. Three measurements were taken. If the differences between three measurements were not greater than 3 mmHg, the second measurement value was considered as the IOP value for that time point. Measurements were performed with cats in sternal recumbency. The head was kept above the level of the heart for each measurement using a soft pillow and the jugular vein was not compressed. STT-1 was measured by placing the commercial strip in the inferior conjunctival fornix for exactly 60 s and reading immediately.

### 2.5 The ERG procedure

A short ERG protocol was recorded from the right eye of each cat using a full-field ERG device (RETevetTM device; LKC Technologies) for veterinary ophthalmology. The ERG protocol was performed by another experienced veterinarian who was blinded to group allocation. The cats were prepared in normal ambient room light. The corneas of right eye were intermittently irrigated with saline to prevent keratopathy, and the eyelids were fixed by a eyelid speculum. Pupil size was assessed frequently to ensure that the pupils were fully dilated during the examination. The flash was placed 15 cm away from the cats. The ERG device included three electrodes: 1. The active electrode, a contact lens with a platinum wire, was placed on the cornea; 2. The reference electrode, a subcutaneous needle electrode (12 mm * 29 gauge), was fully inserted at the bottom of the right ear; 3. Ground electrode, a subcutaneous needle electrode (12 mm * 29 gauge), was fully inserted at the occipital crest. The ERG protocol used in this study was modified from ECVO (European College of Veterinary Ophthalmologists) 5-step single flash Protocol. The specific process was shown in Table A1. The conditions of the photic stimulator without a filter were set at maximum brightness (350 candela s/m^2^). The photopic recordings were conducted 10 min after the cats were administrated with drugs. Then the light was turned off and retinal function was tested after 10 min of dark adaptation.

The implicit times and amplitudes were measured in all ERG responses. Three ERG responses were recorded and the mean values were used for subsequent analysis. The amplitude of a-wave was defined as the difference between baseline and the negative deflection trough. The b-wave amplitude was defined as the difference in amplitude between the a-wave trough and the peak of the b-wave.

### 2.6 Recovery

The effects of dexmedetomidine were reversed with IM atipamezole (12.5 μg/kg; Antisedan, Orion Pharma) for the DEX and DEX + ALF groups 30 min after the treatment. The ALF group was injected with same volume of saline. Subsequent to the experiment, cats received oxygen supplementation for 5 min before being transferred to a temperature-controlled kennel to facilitate recovery.

### 2.7 Data analyses

Data analyses were conducted using SPSS software, version 25 (SPSS Inc., IL, United States), operating on a Windows platform. Normality was assessed by the evaluation of descriptive statistics, plotting histograms and the Shapiro–Wilk test. All age data, weight data, physiological parameters (RT, HR, SAP, DAP and *f*_R_), time metrics (LH, SRr and ST), IOP and STT-1 data were normally distributed. For weight data, time metrics (LH, SRr and ST), and ERG data, means ± standard deviations (SD) were computed and one-way ANOVA with Bonferroni posterior comparisons were performed. For age data, minimum − maximum (median) were computed and one-way ANOVA with Bonferroni posterior comparisons were performed. For physiological parameters (RT, HR, SAP, DAP and fR), IOP and STT-1 data, means ± SD were computed and a two-way repeated measures ANOVA was performed. The statistical significance of the main effects was compared using Bonferroni corrections *post hoc* tests. Statistical significance was set at a *p*-value <0.05.

The sample size was identified through experimental design, pre-experimentation, and conventional numerical settings using PASS 15 (a type 1 error [*p* = 0.05], 90% power). The primary hypothesis of this study was comparing the effects of IM administration of alfaxalone alone or in combination with dexmedetomidine on the short ERG protocol in cats. The preliminary data (unpublished) in four cats is as follow. Under the DA 0.01 in ERG, the b-wave values were 58.6 (DEX + ALF), 50.1 (ALF), 52.37 (DEX), and SDs were 13.61 (DEX + ALF), 15.86 (ALF), 16.67 (DEX) respectively. Based on this, PASS 15 calculated the sample size of 8 animals per group would lead to >90% power using a one-way ANOVA with 5% type 1 error. Hence the use of 8 biological replicates in these experiments for accuracy.

## 3 Result

### 3.1 Basic information

The age and weight data of cats are presented in Table 1. No significant differences were observed in the age or weight comparisons between cats. The sedation procedures previously mentioned were successfully performed in all cats without any adverse events. Furthermore, the sedation effect was sufficient for completing the ERG examination in all three treatments. Vomiting was observed in two cats from DEX group and one cat from DEX + ALF group following IM injection of dexmedetomidine.

**Table 1:** Basic information about the cats in this study.

	Values
Age (months)	3.4 ± 0.6 kg
Weight (kg)	13–25 (18)

Weight data are reported as mean ± SD. Age data are reported as minimum − maximum (median), *n* = 8 cats.

### 3.2 Sedative and reversal times

The times to head-lift (LH), sternal recumbency (SRr), and standing position (ST) were presented in Table 2. Head-lift times were significantly shorter for cats in ALF group compared to those in DEX or DEX + ALF group (*p* < 0.05).

**Table 2:** Times to head-lift (LH), sternal recumbency (SRr), and standing position (ST) after received drugs.

Group	LH (mins)	SRr (mins)	ST (mins)
DEX	30.7 ± 9.6 * (*p* = 0.019)	36.1 ± 4.1	38.2 ± 3.3
ALF	21.8 ± 3.8 *#	35.0 ± 5.3	47.1 ± 11.5
DEX + ALF	37.6 ± 11.4 # (*p* = 0.001)	42.0 ± 10.7	46.8 ± 11.6

DEX + ALF, the group received a combination of dexmedetomidine (5 μg/kg) and alfaxalone (5 mg/kg) intramuscularly; DEX, the group received dexmedetomidine (5 μg/kg) intramuscularly; ALF, the group received alfaxalone (5 mg/kg) intramuscularly, *n* = 8 cats; data are reported as mean ± SD. *Significantly different between the value of DEX and ALF (*p* < 0.05). #Significantly different between the value of DEX + ALF and ALF (*p* < 0.05).

### 3.3 Physiological variables

**Figure 2:**
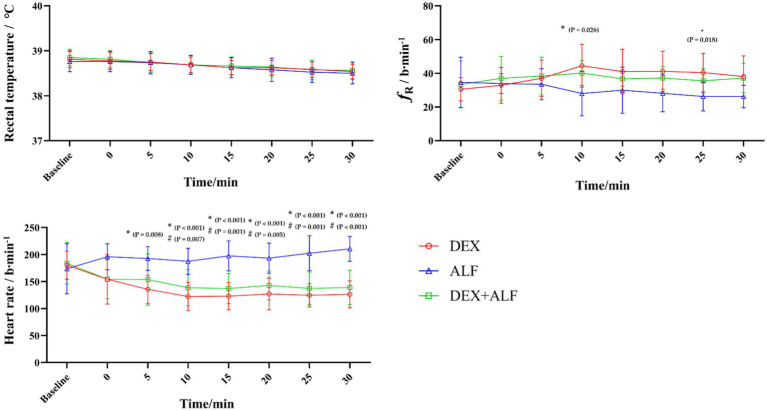
The results of rectal temperature (RT) Respiratory rate (*f*_R_), heart rate (HR) after received drugs. DEX + ALF, the group received a combination of dexmedetomidine (5 μg/kg) and alfaxalone (5 mg/kg) intramuscularly; DEX, the group received dexmedetomidine (5 μg/kg) intramuscularly; ALF, the group received alfaxalone (5 mg/kg) intramuscularly, *n* = 8 cats; data are reported as mean ± SD. Baseline values were measured 30 min before the administrated of drugs. Baseline represents baseline values; T0 represents the injection of drugs, the following timepoints are represent as T5, T10, T15, and so on. *Significantly different between the value of DEX and ALF (*p* < 0.05); #Significantly different between the value of DEX + ALF and ALF (*p* < 0.05); †Significantly different between the value of DEX + ALF and DEX (*p* < 0.05).

**Figure 3:**
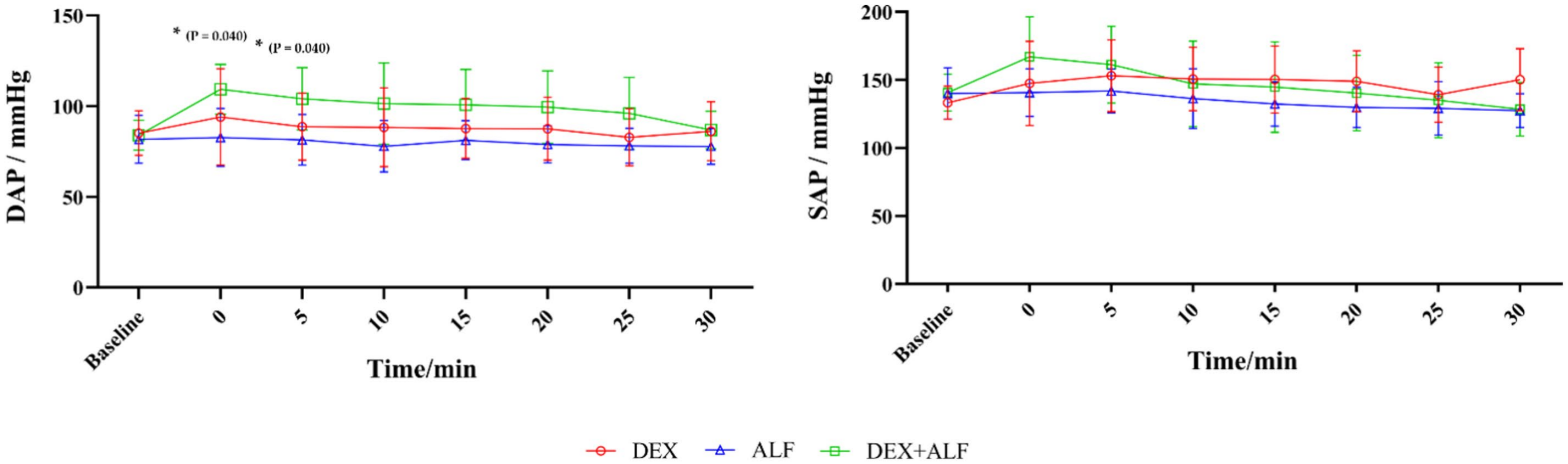
The results of systolic arterial pressure (SAP) and diastolic arterial pressure (DAP) after received drugs. DEX + ALF, the group received a combination of dexmedetomidine (5 μg/kg) and alfaxalone (5 mg/kg) intramuscularly; DEX, the group received dexmedetomidine (5 μg/kg) intramuscularly; ALF, the group received alfaxalone (5 mg/kg) intramuscularly, *n* = 8 cats; data are reported as mean ± SD. Baseline values were measured 30 min before the administrated of drugs. Baseline represents baseline values; T0 represents the injection of drugs, the following timepoints are represent as T5, T10, T15, and so on. *Significantly different between the value of DEX and ALF (*p* < 0.05); #Significantly different between the value of DEX + ALF and ALF (*p* < 0.05); †Significantly different between the value of DEX + ALF and DEX (*p* < 0.05).

The RT, *f*_R_ and HR values were presented in Figure 2. The RT values decreased slightly over time after the drug injection, but remained above 38°C throughout the process in all groups. There were no significant differences in RT values at any time point between the three groups. The HR values increased in ALF group and decreased in DEX and DEX + ALF groups after the injection of drugs. The HR values in ALF group were significantly higher than DEX and DEX + ALF groups at all time points except for baseline, T0, and T5 (*p* > 0.05). ECG monitoring showed no abnormalities during sedation in all three groups. The *f*_R_ was significantly lower at T10 (*p* = 0.026) and T25 (*p* = 0.018) in DEX group compared to ALF group. Compared with DEX and ALF groups, the *f*_R_ showed no significant differences in DEX + ALF group and was over 20 b/min during the study. The SAP and DAP values were presented in Figure 3. There were no significant differences in SAP and DAP between three groups, except for DAP between DEX and ALF groups at T0 (*p* = 0.040) and T5 (*p* = 0.040).

### 3.4 IOP and STT-1

All cats could successfully complete the baseline values of IOP and STT-1 examinations. The results of IOP and STT-1 were presented in Table 3. The IOP values of the right and left eye that measured before this study were 21.79 ± 2.78 and 21.38 ± 2.99 mmHg, respectively. Meanwhile, the STT-1 values of the right and left eye were 16.50 ± 3.11 and 17.12 ± 4.10 mm/min. There were no significant differences in the values of IOP or STT-1 between the left and right eye before treatment (*p* > 0.05). In this trial, IOP and STT-1 examinations were only measured on left eye after administration of drugs.

**Table 3:** The results of intraocular pressure (IOP) and Schirmer tear test 1 (STT-1) after received drugs.

Time	IOP (mmHg)			STT-1 (mm/min)		
	DEX	ALF	DEX + ALF	DEX	ALF	DEX + ALF
Baseline	21.00 ± 3.74	20.62 ± 2.88	22.50 ± 2.20	15.00 ± 4.00	19.25 ± 4.59	17.12 ± 2.80
T0	22.75 ± 3.69	22.25 + 3.65	22.12 ± 2.64			
T5	23.88 ± 4.55	25.25 ± 4.40	23.00 ± 3.42	12.62 ± 3.62	13.62 ± 4.75 ☆ (*p* = 0.010)	12.50 ± 2.78 ☆ (*p* = 0.043)
T10	24.62 ± 3.74	25.62 ± 2.77 ☆ (*p* = 0.009)	22.25 ± 3.28			
T15	26.62 ± 7.05 ☆ (*p* = 0.044)	24.00 ± 2.98	22.88 ± 3.93	7.00 ± 3.25 ☆ (*p* < 0.001)	7.00 ± 2.27 ☆ (*p* < 0.001)	7.12 ± 2.03 ☆ (*p* < 0.001)
T20	25.62 ± 4.56	24.88 ± 2.75	22.88 ± 5.33			
T25	26.12 ± 3.18	24.75 ± 2.49	23.12 ± 6.20	3.62 ± 1.68 ☆ (*p* < 0.001)	2.00 ± 1.31 ☆ (*p* < 0.001)	2.88 ± 1.88 ☆ (*p* < 0.001)
T30	24.50 ± 4.57	24.88 ± 2.95 ☆ (*p* = 0.031)	22.75 ± 4.56			

DEX + ALF, the group received a combination of dexmedetomidine (5 μg/kg) and alfaxalone (5 mg/kg) intramuscularly; DEX, the group received dexmedetomidine (5 μg/kg) intramuscularly; ALF, the group received alfaxalone (5 mg/kg) intramuscularly, *n* = 8 cats; data are reported as mean ± SD. Baseline values were measured 30 min before the administrated of drugs. Baseline represents baseline values; T0 represents the injection of drugs, the following timepoints are represent as T5, T10, T15, and so on. ^☆^Significantly different from the value of baseline.

IOP data from the left eyes did not differ significantly among three groups at any time point (*p* > 0.05). There were no significant differences in IOP measurements in all three groups except T15 (*p* = 0.044) in DEX group, T10 (*p* = 0.009) and T30 (*p* = 0.031) in ALF group compared to the baseline values of each group.

STT-1 data from left eyes did not differ significantly among three groups at any time point (*p* > 0.05). STT-1 values reduced significantly in all three groups during the process except T5 (*p* = 0.784) in DEX group compared to the baseline values of each group.

### 3.5 ERG data

**Figure 4:**
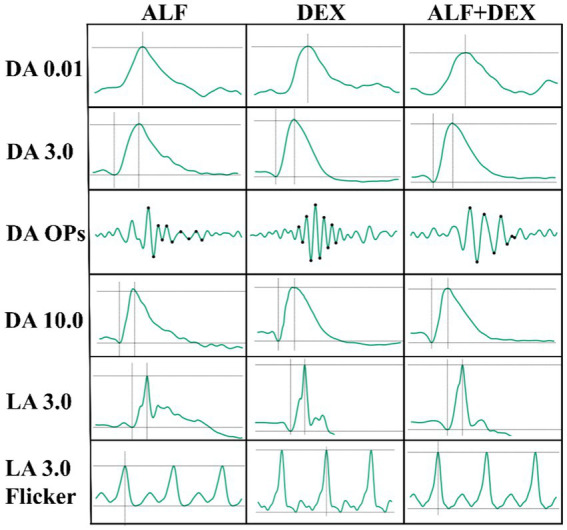
Representative electroretinogram response after received drugs. DEX + ALF, the group received a combination of dexmedetomidine (5 μg/kg) and alfaxalone (5 mg/kg) intramuscularly; DEX, the group received dexmedetomidine (5 μg/kg) intramuscularly; ALF, the group received alfaxalone (5 mg/kg) intramuscularly, *n* = 8 cats. DA 0.01, dark-adapted rod response; DA 3.0, dark-adapted, mixed, rod and cone response; DA Ops, dark-adapted oscillatory potentials; DA 10.0, dark-adapted mixed, rod and cone response to a higher intensity flash; LA 3.0, light-adapted cone response; LA 3.0 flicker, light-adapted 3.0 flicker.

The results of ERG were presented in Table 4 and representative electroretinogram response was shown in Figure 4. ERG data from right eyes had no significant differences among three groups at any time point (*p* > 0.05) except for b-wave amplitude in the condition of DA 0.01 between DEX and ALF groups (*p* = 0.031), a-wave amplitude in the condition of LA 3.0 between ALF and DEX + ALF groups (*p* = 0.008).

**Table 4:** The results of the short electroretinogram protocol after received drugs.

		Animal group		
		DEX	ALF	DEX + ALF
DA 0.01				
b-wave	Amplitude	55.50 ± 27.34 * (*p* = 0.031)	45.85 ± 19.41 * (*p* = 0.031)	60.19 ± 11.26
	Implicit time	48.76 ± 4.80	56.99 ± 7.58	54.42 ± 4.69
DA 3.0				
a-wave	Amplitude	−70.94 ± 31.83	−63.35 ± 33.24	−53.11 ± 29.48
	Implicit time	11.54 ± 0.79	12.71 ± 2.03	11.24 ± 1.36
b-wave	Amplitude	458.25 ± 109.59	366.13 ± 123.34	442.50 ± 160.79
	Implicit time	34.95 ± 3.29	38.16 ± 7.93	38.85 ± 3.77
DA OPs				
	Amplitude	97.30 ± 60.33	90.10 ± 54.56	93.03 ± 33.31
	Implicit time	142.94 ± 24.53	128.61 ± 35.15	143.30 ± 13.85
DA 10.0				
a-wave	Amplitude	−93.03 ± 39.66	−68.29 ± 62.93	−111.63 ± 63.46
	Implicit time	9.98 ± 1.01	10.91 ± 2.10	10.04 ± 1.91
b-wave	Amplitude	483.38 ± 125.32	415.00 ± 134.98	459.13 ± 160.92
	Implicit time	32.85 ± 2.35	35.68 ± 6.12	34.21 ± 3.23
LA 3.0				
a-wave	Amplitude	−7.00 ± 1.53	−5.29 ± 2.23 # (*p* = 0.008)	−8.90 ± 2.47 # (*p* = 0.008)
	Implicit time	8.88 ± 0.49	10.41 ± 1.63	9.84 ± 1.90
b-wave	Amplitude	71.64 ± 22.37	70.16 ± 33.69	62.78 ± 21.83
	Implicit time	22.48 ± 5.67	29.99 ± 9.63	23.01 ± 6.29
LA 3.0 flicker				
	Amplitude	78.66 ± 34.51	47.11 ± 17.97	56.38 ± 21.59
	Implicit time	36.93 ± 18.39	24.28 ± 12.16	24.01 ± 12.04

DEX + ALF, the group received a combination of dexmedetomidine (5 μg/kg) and alfaxalone (5 mg/kg) intramuscularly; DEX, the group received dexmedetomidine (5 μg/kg) intramuscularly; ALF, the group received alfaxalone (5 mg/kg) intramuscularly, *n* = 8 cats; data are reported as mean ± SD. *Significantly different between the value of DEX and ALF (*p* < 0.05); ^#^Significantly different between the value of DEX + ALF and ALF (*p* < 0.05).

## 4 Discussion

Compared with ophthalmoscopic and behavioral examinations, the ERG allows for an objective characterization of specific cell types in the retina and can be used for earlier diagnosis of retinal disease (29). The administration of anesthetic drugs in animals can avoid background noises generated by body or globe movements during ERG recording. It is crucial to regulate IOP and STT-1, especially IOP, to ensure the success of ophthalmic surgery (30), however, there are few reports about the effects of alfaxalone on ocular parameters in cats. Therefore, we evaluated the effect of IM administration of DEX, ALF, or DEX + ALF groups on short ERG protocol, IOP, and STT-1 in cats. All three treatments enabled the cats to tolerate corneal electrodes and subcutaneous needle electrodes. After the reversal with atipamezole in DEX and DEX + ALF groups, head-lift times were significantly shorter than ALF group. The time to recovery from sedation had no significant differences in three treatments.

The short ERG protocol used in this study includes the mixed, rod, and cone responses to light or dark conditions. Three treatments allowed for the successful completion of the ERG examination in cats. ERG changed with different sedation treatments. A higher a-wave amplitude under light-adapted cone response (LA 3.0) conditions was found in DEX + ALF group compared to ALF group, which indicated a difference in the overall assessment of the outer and inner retina in the day vision. Similar to the findings in this study, a previous study observed significant differences in the photopic b-wave amplitude when ketamine was used combined with xylazine or dexmedetomidine in cats (31). And a higher b-wave amplitude under dark-adapted rod response (DA 0.01) conditions was found in DEX group compared to ALF group, which indicated a difference in the rod responses in night vision. Other ERG variables showed no significant changes among three treatments, including: 1. Light-adapted flicker (LA 3.0 flicker) evoked a robust response of the cone pathway; 2. Dark-adapted, mixed, rod and cone response (DA 3.0) that assessed both rod and cones; 3. Dark-adapted mixed, rod and cone response to a higher intensity flash (DA 10.0) that evaluated the retinal function on the media opacity; 4. Dark-adapted oscillatory potentials (DA OPs) extracted from the mixed response by using the 85 Hz bandpass filter, providing more subtle and consistent information about the diffused changes in the inner retina caused by ischemic events or amacrine cell function. Awake cats cannot perform ERG protocol in this trail. But studies have shown that anesthesia and sedation resulted in significant attenuation and delay of ERG responses in dogs (6).

Local anesthesia and mydriasis might potentially influence the measurements of IOP and STT-1, tropicamide and local anesthetic drugs were not used in the left eye in this trial (32–34). There were no significant differences in IOP and STT-1 values between the left and right eyes before the start of sedation, and it had been reported that there were no significant differences between IOP and STT in the left and right eyes after anesthesia (14). Thus we conducted the IOP and STT-1 measurements exclusively on the left eye. Alfaxalone (1–3 mg/kg IM) is reported to significantly increase IOP in dogs that were premedicated with dexmedetomidine or without any premedication (35). Wolfran et al. reported that IM administration of dexmedetomidine (10 μg/kg) reduced IOP and tear production in cats, while a lower dose of 7.5 μg/kg had no significant effect on IOP (36). In dogs receiving intravenous injection of 5 μg/kg dexmedetomidine, there was no significant difference in IOP measurements within 10 min of drug administration (37, 38). Cats sedation with intramuscular 100 μg/kg medetomidine did not cause a statistically significant change in IOP values (14). In this study, we only observed statistical differences at T15 in DEX group, T10, and T30 in ALF group compared to their baseline, respectively. The lower dose of dexmedetomidine (5 μg/kg) used in our study might explain the discrepancy with the previous study. Additionally, DEX + ALF group could potentially counteract the elevation of IOP compared to ALF group. It was speculated to be related to changes in the production and outflow of aqueous humour (39). But further research is needed to investigate the underlying mechanisms. Corneal injuries are common ocular complications due to the lack of eyelid protection and deficiency of tear production (40). Previous studies demonstrated that medetomidine-ketamine, dexmedetomidine, methadone, dexmedetomidine-methadone and general anaesthesia could reduce tear production (12, 36, 41). Similarly, in the present study, all three treatments reduced tear production in cats.

Notably, even at the lower dose utilized in this study, IM administration of dexmedetomidine (5 μg/kg) resulted in a reduction in HR in cats from the DEX and DEX + ALF groups. These findings are consistent with prior studies indicating that dexmedetomidine administration results in decreased HR in cats, irrespective of dose (26, 42, 43). Alfaxalone treatment demonstrated minimal cardiovascular depression at clinical routine doses (2–10 mg/kg). Previous studies showed that administering supraclinical doses of alfaxalone alone (15 and 50 mg/kg) can lead to dose-dependent decrease in HR and arterial blood pressure in cats (21). The increase in HR is observed after the IM administration of various doses of alfaxalone (1, 2.5, 5, 10 mg/kg) (44). The results of the present trial are similar to these studies, the HR increased following the IM administration of alfaxalone in ALF group. SAP and DAP values displayed a biphasic pattern in DEX + ALF and DEX groups, with blood pressure values initially increased for the first 10 min after the treatment, subsequently declining gradually to baseline levels. It may be due to the alpha-2 adrenergic agonists such as dexmedetomidine can induce peripheral vasoconstriction by activating post-synaptic alpha-2 receptors in the peripheral vascular smooth muscle, leading to an increase in blood pressure. Blood pressure may subsequently decrease due to a central effect on sympathetic tone (45). The administration of alfaxalone (5 mg/kg, IM) and dexmedetomidine (10, 20, and 40 μg/kg, IM) also showed similar results on arterial blood pressure in cats (26, 46). The administration of alfaxalone in cats caused a dose-dependent suppressive effect on arterial blood pressure (21, 47). In this study, the blood pressure showed a gradual decrease and stabilized after 15 min, no significant suppression was recorded after the administration of alfaxalone.

In the present study, apnea was not observed and *f*_R_ was well maintained in all treatments. Apnea after the IV administration of alfaxalone has been reported as an uncommon side effect in cats (47). The IM administration of 10 μg/kg dexmedetomidine and 5 mg/kg alfaxalone with or without 0.2 mg/kg butorphanol in cats has been reported to decrease the respiratory rate which was not observed in this study (46, 48). This might be related to the lower doses of drugs used in the present work.

Some limitations in this study should be noted. Firstly, the relatively small sample size may affect the generalizability of the findings. Furthermore, baseline data of ERG was not obtained as a blank control in cats. Finally, the cats used in this study were young, healthy, spayed female cats, and older, sick, or male cats may respond differently to these treatments.

## 5 Conclusion

In the present study, we successfully completed the short ERG examination under three protocols in cats. Except for higher photopic a-wave amplitude in DEX + ALF group compared with ALF group, no significant changes in the ERG variables were observed in three treatments. All these treatments resulted in increased IOP values and reduced STT-1 values.

## Data availability statement

The original contributions presented in the study are included in the article/Supplementary material, further inquiries can be directed to the corresponding author.

## Ethics statement

The animal study was approved by the Institutional Animal Care and Use Committee of Nanjing Agricultural University. The study was conducted in accordance with the local legislation and institutional requirements.

## Author contributions

YG: Conceptualization, Data curation, Investigation, Methodology, Visualization, Writing – original draft. SM: Writing – review & editing. ZZ: Writing – review & editing, Project administration, Supervision.
