# Supplementary material for: Effects of intramuscular alfaxalone and dexmedetomidine alone and combined on ocular, electroretinographic, and cardiorespiratory parameters in normal cats
Source: Front Vet Sci. 2024 Jul 3;11:1407928. doi: 10.3389/fvets.2024.1407928 (PMC11251925; doi:10.3389/fvets.2024.1407928)
Supplement: Supplementary file 1 [file Table_1.DOCX]

**Appendix**

**TABLE A1**┃The electroretinogram protocols used in this study, which was adapted from ECVO (European College of Veterinary Ophthalmologists) 5-step single flash guidelines.

| **Description** | **Time** | **Flash luminance energy**  **(0.33, 0.33 white)** | **Background luminance**  **(0.33, 0.33 white)** | | **Flashes** |
| --- | --- | --- | --- | --- | --- |
| Light adaptation | 10 mins |  | | 30 cd/m² |  |
| Light-adapted cone response (LA 3.0) |  | 3 cd·s/m², 2 Hz | | 30 cd/m² | 20 |
| Light-adapted 3.0 flicker (LA 3.0 flicker) |  | 3 cd·s/m², 28.3 Hz | | 30 cd/m² | 141 – 424 |
| Dark adaptation | 10 mins |  | | Off |  |
| Dark-adapted rod response (DA 0.01) |  | 0.01 cd·s/m², 0.2 Hz | | Off | 1 |
| Dark-adapted, mixed, rod and cone response (DA 3.0) |  | 3 cd·s/m², 1/15 Hz | | Off | 1 |
| dark-adapted oscillatory potentials (DA OPs) |  | 3 cd·s/m², 85 Hz | | Off | 141 – 424 |
| Dark-adapted mixed, rod and cone response to a higher intensity flash (DA 10.0) |  | 10 cd·s/m², 0.05 Hz | | Off | 1 |
